# Therapeutic Potential of *Carica papaya* (L.) Extract on NRF2/KEAP1 and Apoptotic Pathways in Asbestos‐Induced Lung Toxicity

**DOI:** 10.1002/fsn3.71984

**Published:** 2026-06-29

**Authors:** Usman Haider, Muhammad Ahmad, Wania Nasir, Najeeb Ullah Khan, Muhammad Saad Tariq, Bilal Aslam, Saif Ur Rehman, Junming Sun

**Affiliations:** ^1^ Institute of Physiology and Pharmacology, Faculty of Veterinary Science University of Agriculture Faisalabad Faisalabad Pakistan; ^2^ Institute of Animal Science Chinese Academy of Agricultural Sciences Beijing China; ^3^ Department of Physiology The University of Faisalabad Faisalabad Pakistan; ^4^ Department of Rehabilitation Sciences The University of Faisalabad Faisalabad Pakistan; ^5^ Department of Public Health Rawalpindi Medical University Rawalpindi Pakistan; ^6^ Laboratory Animal Centre Guangxi Medical University Nanning Guangxi China

**Keywords:** alveolar architecture, antioxidant defense, differential oxidant profiling, irritant‐induced toxicity, mitochondrial apoptosis, plant‐derived compounds, pulmonary toxicity

## Abstract

Plant derived natural products and medicinal herbs have recently gain more attention as therapeutic agents to treat various ailments. The current study aims to explore the potential role of *C. papaya* 's natural intervention in reducing the asbestos‐induced pulmonary damage. A total of 32 male albino rats were grouped into NC, PC, STD (Montelukast Sodium 10 mg/kg), and C.P (500 mg/kg) groups, having eight animals in each group. Experimental units from the PC were exposed to asbestos fibers in a glass chamber for 2–3 h daily. Biological tissues for histopathology were preserved in formalin, lung tissue for qRT‐PCR was immersed in TRIzol, broncho‐alveolar lavage fluid (BALF) was collected for protein analysis, and serum was separated for oxidative biomarkers. Data was analyzed by the ANOVA and Tukey's test, and GraphPad Prism (v 8.0) was used for graphical representation. Results of the study revealed that after treatment, serum antioxidant markers were significantly enhanced in the C.P group *(p ≤ 0.05)*, whereas oxidative markers were reduced significantly in the C.P group *(p ≤ 0.05)*. Markers of protein analysis from BALF and wet‐to‐dry ratio increased in the PC group, but a significant decline was seen in the C.P group *(p ≤ 0.05)*. Results of qRT‐PCR showed upregulation of oxidative response pathway molecules and apoptotic markers in PC such as NRF1, DUOX, DUOXA1, DUOXA2, BAX, BID, CASP9, CYT‐C, and NFE‐2 L2. Parallel to this, an elevated response from antioxidant markers and calcium homeostasis regulators was seen in the C.P‐treated group. Microscopic insights revealed normal parenchyma of the lung and trachea in the NC group, which was worsened with thickened airways, fibrotic alveolar walls, and lodged fibers of asbestos seen in the tissue samples of the PC group. Treatment with *C. papaya* revealed a restored histopathological profile with better alveolar architecture and mitigated effects of asbestos fibers. The results of this study suggest that *C. papaya* exhibits strong antioxidant potential to mitigate oxidative stress caused by inorganic source.

## Graphical abstract

Asbestos exposure induced oxidative stress, apoptosis, and lung tissue damage in animal models. Treatment with *Carica papaya* (500 mg/kg) extract improved antioxidant status, reduced oxidative stress biomarkers, and restored lung architecture. Gene expression analysis showed modulation of NRF2/KEAP1 and apoptotic pathways. The findings indicate the protective role of *C. papaya* against asbestos induced pulmonary toxicity.

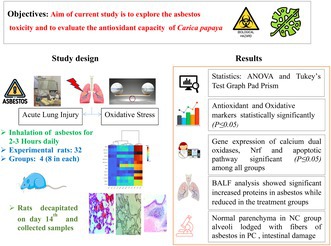

**Abbreviations**
BAD: BCL2‐associated agonist of cell death
BALF: Bronchoalveolar lavage fluid
BAX: Bcl2 associated X
BCL2: B‐cell lymphoma 2
BID: BH3 Interacting Domain Death Agonist
CALM‐2: Calmodulin −2
CASP9: Caspase 9
CYT‐C: Cytochrome C

*C. papaya*

:
*Carica papaya*

DUOX: Dual Oxidases
DUOXA1: Dual oxidase maturation factor 1
DUOXA2: Dual oxidase maturation factor 2
GRK‐2: G‐protein coupled receptor Kinase 2
KEAP‐1: Kelch like ECH associated Protein 1
NFE2L2: Nuclear Factor, Erythroid 2 Like 2 (NRF2)
NRF1: Nuclear factor erythroid 2–related factor 1
PIAS‐2: Protein inhibitor of activated STAT 2
qRT‐PCR: Quantitative Real‐time Polymerase Chain Reaction

## 1 Introduction

Acute lung injury (ALI) is characterized by an inability of the lungs to exchange gases, which may be due to smoke, pulmonary infections, and inorganic irritant particles such as asbestos and carbon (Lagarde et al. 2024; Xiao et al. 2025). Asbestos is commonly found in construction materials, shipping applications, household items, and industrial items, and exposure to its fibers can pose a significant threat to health. The extent of severity of symptoms after exposure depends on factors such as duration, variable physical nature, and individual characteristics. Fibers that are thin, long, and have a high aspect ratio are particularly hazardous and deeply penetrate the lungs (Gong et al. 2024). Due to its fibrous microscopic nature, asbestos induces cellular damage at the bronchiolar alveolar interface, prompting epithelial cell damage and macrophage activation, causing asbestosis (Wen et al. 2023). These fibers can trigger the upregulation of genes that control growth factors, mediating cell proliferation and excessive connective tissue deposition (Iftikhar et al. 2015). Individual factors like genetics, respiratory rate, and age can influence susceptibility. Stringent regulations and safety measures are crucial for minimizing exposure and safeguarding public health (Sahin and Koksal 2023). Chronic obstructive pulmonary disease (COPD) is an inflammatory lung disease that causes restricted airflow and breathing problems, including emphysema, chronic bronchitis, or small airway obstruction (Wang et al. 2024). Patient's clinical conditions progressively deteriorated, presenting complications such as progressive infection, leukopenia, liver dysfunction, electrolyte imbalances, and respiratory alkalosis (Yang et al. 2025).

Oxidative stress can be triggered by several factors, including diabetes, cancer, obesity, cardiovascular disorders, exposure to heavy metals, UV‐rays, and environmental factors (Chung et al. 2024). Physiologically reactive nitrogen and reactive oxygen species (RNS and ROS) have a role in cell signaling, metabolism, inflammation, transmission of nerve impulses, respiration, healing of wounds, and dilation of the blood vessels (Day 2024). Biological reactive species include superoxide anions, hydrogen peroxide, and hydroxyl radicals, which are released in response to lipid peroxidation, DNA damage, and phagocytic activity (Guo et al. 2021; Tejchman et al. 2021). There is an antioxidant mechanism in the body that is always active to remove excessive free radicals and maintain redox homeostasis. These include superoxide dismutase (SOD) and glutathione peroxidase (GPx), which are produced in the mitochondria, and catalase (CAT), which is generated from the peroxisomes (Fernando et al. 2024). These cause the conversion of the hydroxyl radicals into hydrogen peroxide, which is then further dissociated into water and oxygen molecules (Liu et al. 2022).

In recent years, various plants and their derived bioactive compounds have been used as a complementary and alternative medicine to cure infectious and non‐infectious diseases (Bagheri et al. 2024). Plants enriched in bioactive compounds have therapeutic potential and are now gaining attention. In Pakistan, *C. papaya* is traditionally used in dengue fever, wound healing, and liver disorders as an anti‐inflammatory agent and for boosting immunity (Hariono et al. 2021). Generally, leaves, bark, fruit, and latex extracted from this plant are used as herbal medicine, but it has been shown that leaves are rich in papain, flavonoids, alkaloids, saponins, ascorbic acid, cystatin, cyanogenic glucosides, chymopapain, and minerals (Tan et al. 2021). *C. papaya* is well known for reducing and neutralizing oxidative stress because it is enriched in ascorbic acid, carotenoids, vitamin E, flavonoids (kaempferol and quercetin), phenolic compounds (i. gallic acid, ii. ferulic acid, iii. caffeic acid), and enzymes (including chymopapain and papain) (Munir et al. 2022). These bioactive compounds reduce the peroxidation of lipids, help in scavenging the free radicals, and mitigate oxidative stress due to reactive oxygen species (ROS) (Gudimella et al. 2022). *C. papaya* has endogenous precursors of glutathione and lycopene, which specifically detoxify the ROS and RNS in lungs (Khor et al. 2021). Thus, the current study is aimed to determine the antioxidant efficacy of *C. papaya* by exploring its pharmacological potential and phytochemical characteristics, aligned with asbestos‐induced pulmonary toxicity. The current work addresses the research gap of molecular profiling in asbestos‐induced pulmonary distress coupled with phytochemical therapy, the emphasis being on the impact of *C. papaya* on NRF2/KEAP1, apoptotic signaling pathways, and its impact on BALF.

## 2 Materials and Methods

### 2.1 Study Design

A total of 32 male albino Wistar rats were raised at the animal house facility according to guidelines issued by the Animal Ethics Committee (Office of Research, Innovation, and Commercialization, UAF—DC. No. 1547/ORIC). Leaves of the plant were collected from the botanical garden of University of Agriculture Faisalabad Pakistan, and the herbarium number of the plant (Voucher # 1017–2‐2023) was issued by the Department of Botany, University of Agriculture Faisalabad, Pakistan. Toxicity assay of the extract was done to determine the dose rate according to body weight and behavioral signs observed (Table 1). The experimental units were randomly divided into 4 groups with eight rats in each group. Group 1 was considered the Negative Control group (NC), receiving a normal diet. Group 2 was Positive Control (PC) exposed to asbestos fibers through inhalation in a glass chamber that was connected to the continuous air flow with the pump to disperse the asbestos particles and a small inlet on the side of the glass chamber for ventilation for 2–3 h daily up to 2 weeks (Haider et al. 2025). Group 3 was the Standard Treatment group (STD) in which standard drug Montelukast sodium at a dose rate of 10 mg/kg body weight was administered orally, along with daily asbestos exposure. Group 4 was considered the Herbal Treatment group (C.P), exposed daily to asbestos and treated with ethanolic extract of *C. papaya* leaves at a dose rate of 500 mg/kg body weight orally. The single dose of plant extract was selected on the basis of toxicity assay and ADMET profile of the extract, performed and validated in our previous study (Haider et al. 2025; Haakonde et al. 2026). Experimental units were euthanized on day 14, and biological samples for serology, histopathology, BALF analysis, and qRT‐PCR were collected.

**TABLE 1:** Behavioral analysis during acute toxicity assay.

Parameters	0.5 h	24 h	48 h	4 days	14 days
Diarrhea	Absent	Absent	Absent	Absent	Absent
Respiration	Normal	Normal	Normal	Normal	Normal
Eye move	Normal	Normal	Normal	Normal	Normal
Allergy	Absent	Absent	Absent	Absent	Absent
Sleep	Normal	Normal	Normal	Normal	Normal
Nervous sign	Absent	Absent	Absent	Absent	Absent
Mortality	Nil	Nil	Nil	Nil	Nil
Behavior	Normal	Normal	Normal	Normal	Normal

*Note:* Control and ethanolic group.

### 2.2 Plant Extract Analysis

#### 2.2.1 Plant Extraction Protocol

Leaves of the *C. papaya* were collected from the botanical garden of University of Agriculture Faisalabad, Pakistan, then they were subjected to shade drying. The leaves were crushed and ground into fine powder. From the total obtained powder, 500 g powder was weighed and mixed with 1500 mL of 70% v/v ethanol. The subsequent solution was allowed to soak for 48–72 h. Filtration was done afterwards, and the filtrate was subjected to the rotary evaporator. The obtained material was placed in an incubator at 60°C for drying, and the resultant pure extract was stored in air tight falcon tubes (Ali 2024).

### 2.3 Serum Oxidative Stress and Antioxidant Levels

Blood was collected in a gold‐topped vacutainer and then centrifuged at 5000 rpm for five minutes to collect the serum. SOD was measured using a commercially available SOD activity assay kit (Elabscience—catalog no. BC0175). CAT activity was determined using assay kit (Elabscience—catalog no. BC0205). GSH‐Px (GPX glutathione peroxidase) activity was analyzed using the assay kit (Elabscience catalog no. E‐BC‐K096‐M). GST (Glutathione S‐Transferase) was done using a Glutathione S‐Transferase assay kit (Elabscience—catalog no. E‐BC‐K278‐S) (Rasheed et al. 2025). Total antioxidant capacity (TAC) was detected by the calorimetric kit (Elabscience—catalog no. E‐BC‐K801‐M). Serum MDA (Malondialdehyde) was measured by TBRAS assay kit (catalog no. E‐BC‐K298‐M). NO (Nitric Oxide) was measured with a calorimetric commercial kit (Elabscience—catalog no. E‐BC‐KO35‐35). Total Oxidative Stress (TOS) was determined utilizing a calorimetric assay kit (catalog no. E‐BC‐K802‐M). Iron (Fe) content was determined with the help of a commercial assay kit (Elabscience kit catalog no. E‐BC‐K139‐S).

### 2.4 Broncho‐Alveolar Lavage Fluid (BALF)

Lungs were bathed with chilled saline using a sterile nasogastric tube. The BALF was collected after three retractions in an Eppendorf tube and centrifuged at 3 g (4°C) and stored at −20°C. BALF was used for the determination of lactate dehydrogenase, albumin content, total protein, and glycoprotein (Bazzano et al. 2020).

### 2.5 Wet‐To‐Dry Ratio

Immediately after the extraction, the lungs were weighed and then placed in an incubator for 3 days at 60°C. After incubation, the lobes were weighed again, and the difference in fluid content before and after incubation was noted (Ge et al. 2023).

### 2.6 Gene Expression Analysis

Total RNA was extracted from TRIzol‐preserved lung tissues, and cDNA synthesis was done using Revert Aid first‐strand cDNA kit (Ref. No.: K1622 LOT.01169153 Thermo Scientific). Maxima SYBR Green/ROX qPCR Master mix (2X) (Ref No.: K0221 LOT.01134497) and 0.1 mL PCR 8‐strip tubes (Cat. No. 403102) were used for gene expression analysis through qRT‐PCR. Data was analyzed by the 2^−ΔΔCt^ method.

### 2.7 Histopathology

Histopathology of the lung tissue and trachea was performed using the formalin‐fixed paraffin‐embedded tissue technique. Tissues of the lungs and trachea were stained with Hematoxylin and Eosin (H&E Stain), and Van Gieson's (VG) stain (Slaoui and Fiette 2011).

### 2.8 Statistical Analysis

Experimental data were analyzed by using ANOVA and post hoc Tukey's test to determine the significance between all groups, and graphical representation was done through GraphPad Prism (v 8.0).

## 3 Results

### 3.1 Plant Extracts Analysis

Phytochemical and antioxidant characteristics of *C. papaya* were studied to determine its potential. Total phenolic and total flavonoid compounds are used to quantify the compounds that are significant due to their antioxidant, anti‐inflammatory, and other health‐promoting properties. The antioxidant potential evaluated through DPPH (Free radical scavenging activity) which showed significant inhibition percentage. FRAP (Ferric‐reducing Antioxidant Power assay) was demonstrating the ability of electron donation. Similarly, the TPC was 540 ± 2.8868 mg Gallic acid/g, and TFC was 13.42 ± 1.7321 mg Catechin/g. These results demonstrated the antioxidant potential of the extract and its key role in mitigating oxidative stress as shown in Table 2.

**TABLE 2:** Phytochemical and antioxidant characters of *C. papaya* .

DPPH (inhibition percentage)	69 ± 0.5774
FRAP (mMFe^2^+/g)	11.72 ± 0.1101
TPC (milligram gallic acid per gram)	540 ± 2.8868
TFC (milligram catechin per gram)	13.42 ± 1.7321

### 3.2 Serum Antioxidant Markers

**FIGURE 1:**
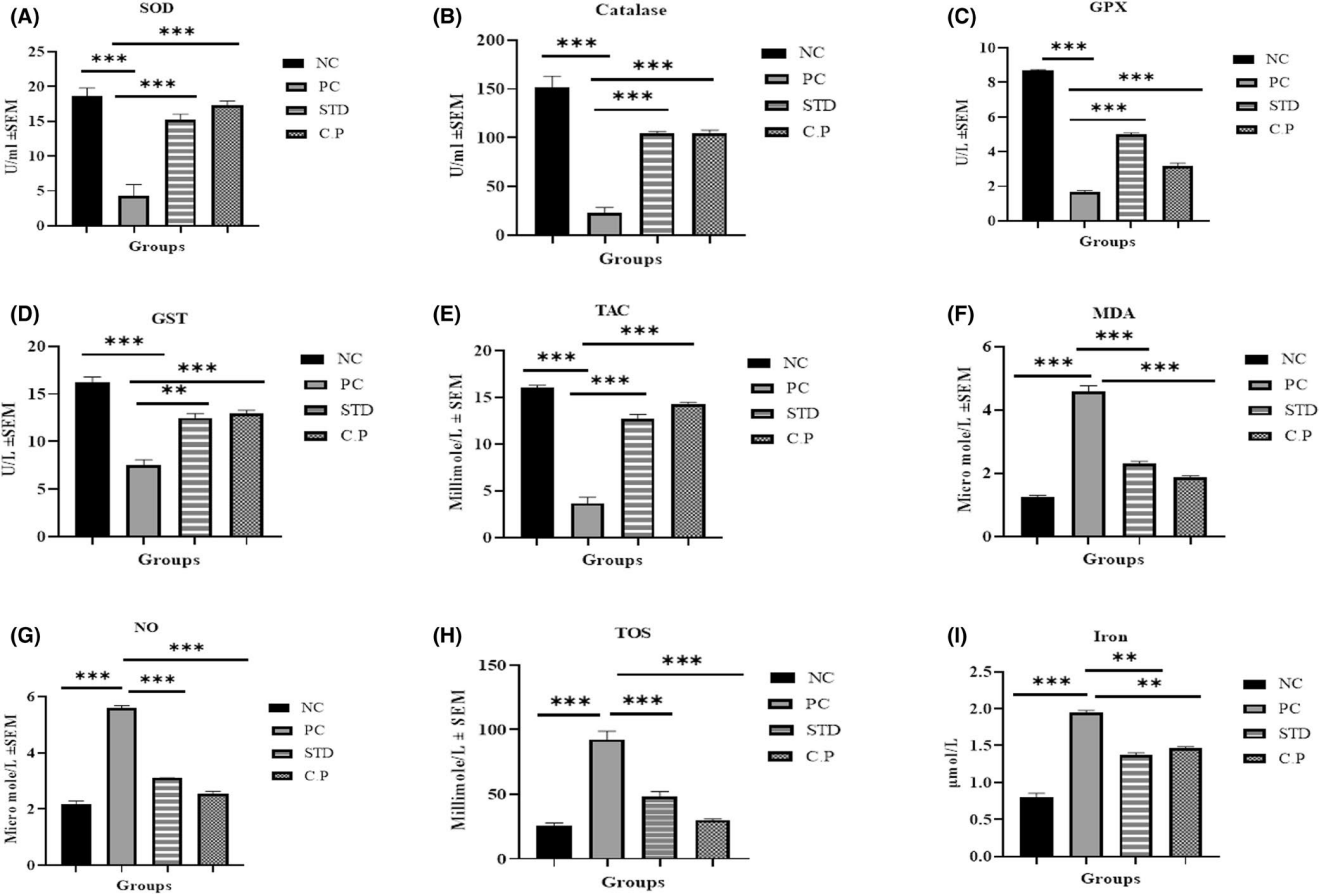
Serum analysis for the biomarkers of oxidative stress and antioxidant status are graphically represented with significant contrast of NC, PC, STD, and C.P groups shown. (A) Represents the serum superoxide dismutase (SOD) with a significant (*p* ≤ 0.05) decrease in the PC group compared to the other groups. (B) Shows Serum Catalase activity having significant (*p* ≤ 0.05) decrease in PC however, increases in other groups. (C) Serum Glutathione Peroxidase (GPX) alteration was seen in NC, PC, STD, and C.P groups. (D) Glutathione‐S‐Transferase (GST) was raised significantly (*p* ≤ 0.05) when compared among all groups. (E) Total antioxidant capacity (TAC) has been shown with a significant (*p* ≤ 0.05) correlation in each group. (F) Malondialdehyde (MDA) level in the serum depicts significant (*p* ≤ 0.05) upheaval in PC in comparison to all groups. (G) Nitric Oxide (NO) level in serum is expressed significantly (*p* ≤ 0.05) increased in PC compared to STD and C.P groups. (H) Level of serum Total oxidative stress (TOS) with statistical description among all groups shows significant (*p* ≤ 0.05) increase in PC group. (I) Changes in iron concentration indicate a significant increase (*p* ≤ 0.05) in PC compared to all the other groups. Data was analyzed through one‐way ANOVA and significance was determined by Tukey's Post Hoc Test.

Statistical results of serum SOD revealed a significant decrease in the PC group *(p ≤ 0.05)* as compared to the NC group, whereas a significant increase of serum SOD in the STD and C.P groups *(p ≤ 0.05)* was observed as shown in Figure 1A. Similarly, serum catalase was significantly reduced in the PC group *(p ≤ 0.05)*; however, the levels were restored in the STD and C.P groups, showing statistical significance at *p ≤ 0.05*, as shown in Figure 1B. A consistent trend was also observed in the statistical interpretation of GPx activity, with a significant decrease in the PC group *(p ≤ 0.05)*, while its level was elevated in the STD and C.P group, as demonstrated in Figure 1C. This pattern was also exhibited as a low level of GST in the PC group *(p ≤ 0.05)* in comparison to the NC group, whereas significantly increased values were seen in the C.P and STD groups *(p ≤ 0.05)*, as shown in Figure 1D. Serum TAC showed significant variations in the PC group as compared to the STD and C.P groups shown in Figure 1E. Collectively, the obtained results demonstrate the impairment of antioxidant markers in the PC group, while effective restoration was observed due to the therapeutic potential of the *C. papaya* in the C.P group.

### 3.3 Serum Oxidative Stress Markers

Serum oxidative stress was measured to correlate asbestos toxicity with the generation of ROS. MDA activity is related to lipid peroxidation, and in this study, serum MDA levels were significantly increased in the PC group *(p ≤ 0.05)* compared to the NC group. However, a significant reduction in MDA level was observed in C.P and STD groups *(p ≤ 0.05)* as shown in Figure 1F. The results of serum NO exhibited significantly elevated levels in the PC group *(p ≤ 0.05)* compared to the NC group, whereas in the STD and C.P groups, there was a significant reduction in NO level *(p ≤ 0.05)* as shown in Figure 1G. Serum TOS was significantly higher in the PC group *(p ≤ 0.05)* in comparison to the NC group while it was significantly decreased in the STD and C.P groups *(p ≤ 0.05)* as depicted in Figure 1H. Fe contributes to the generation of oxidative stress through Fenton's reaction. The statistical result of Fe was highly significant in the PC group *(p ≤ 0.05)* compared to the NC group. There was a significant decline in Fe levels in both treatment groups *(p ≤ 0.05)* as graphically presented in Figure 1I. These results provide a coherent pattern of heightened oxidative stress due to asbestos toxicity and the therapeutic potential of *C. papaya* in mitigating the oxidative damage in treated groups.

### 3.4 Broncho‐Alveolar Lavage Fluid Analysis

**FIGURE 2:**
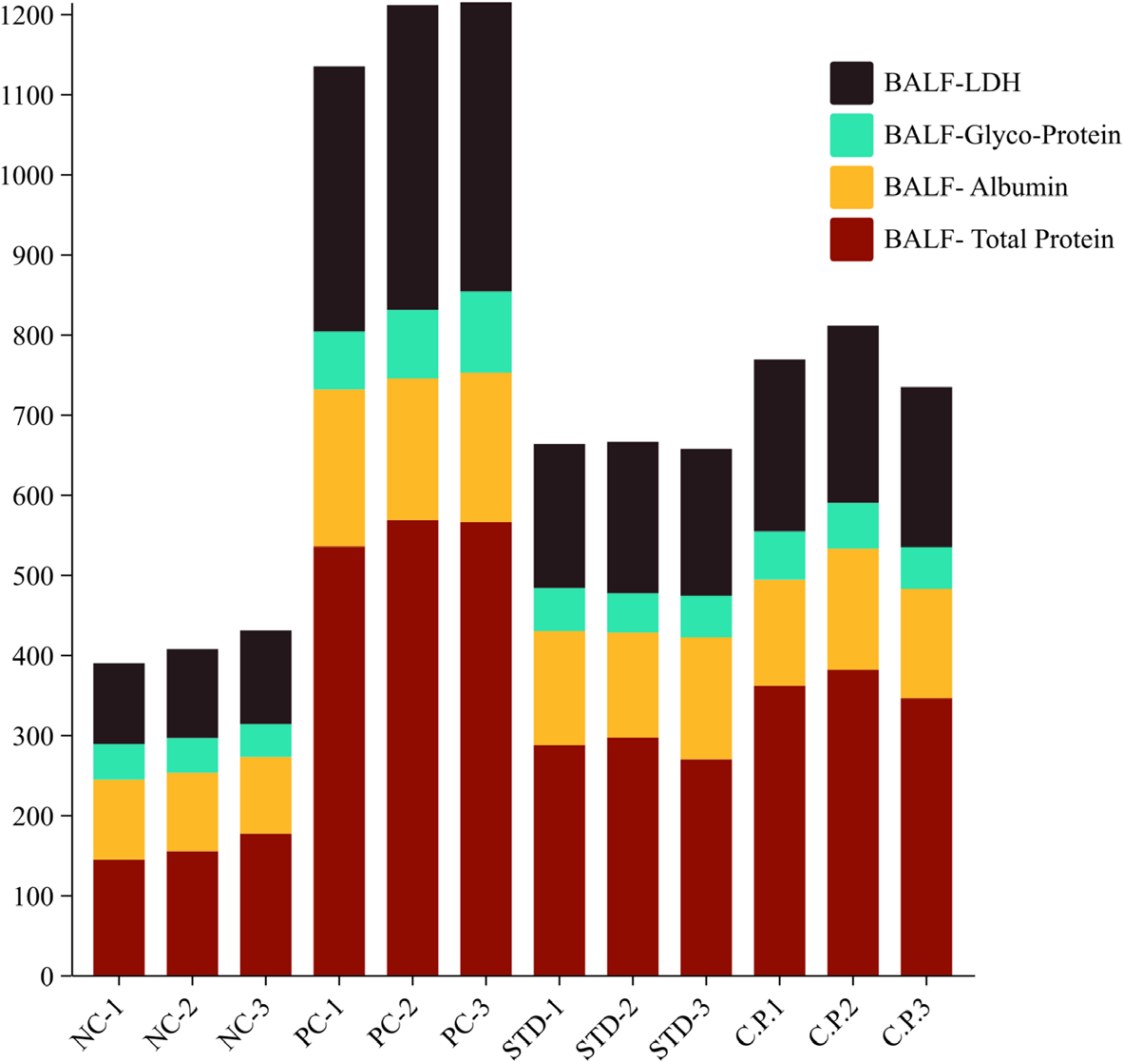
Bronchoalveolar lavage fluid (BALF) revealed significant differences (*p* ≤ 0.05) among the NC, PC, STD, and C.P groups in several parameters. Lactate dehydrogenase (LDH) levels showed significant differences between all groups; albumin protein levels also differed significantly when comparing all groups. Total protein concentration differed significantly among the NC, PC, STD, and C.P groups. Glycoprotein levels also showed significant differences when comparing all groups.

BALF was collected from the lungs and tracheal regions to assess protein leakage and dead cell debris due to tissue damage, reflecting the extent of injury and of therapeutic interventions. LDH is the marker of cellular damage, which was significantly increased in PC *(p ≤ 0.05)* in contrast to the NC group, while a significant decrease was observed in the STD and C.P groups *(p ≤ 0.05)*, respectively, as shown in Figure 2. BALF albumin is an indicator of protein leakage from the membranes. Subsequently, a significant elevation of BALF albumin content was seen in PC *(p ≤ 0.05)* compared to the NC group. There was a significant decline in the BALF albumin content in the STD and C.P group *(p ≤ 0.05)*, as shown in Figure 2. BALF analysis revealed higher total protein levels in the PC group *(p ≤ 0.05)* compared to the NC group. However, this increase was significantly reduced in both treatment groups *(p ≤ 0.05)*, as shown in Figure 2. BALF glycoprotein levels were elevated in the PC group compared to the NC group, but were significantly decreased in the STD and C.P groups *(p ≤ 0.05)* (Figure 2). These findings suggest that asbestos‐induced acute toxicity damages the pulmonary tissue, whereas treatment with *C. papaya* helped restoration of membrane integrity.

#### 3.4.1 Lung Wet‐To‐Dry Ratio

**FIGURE 3:**
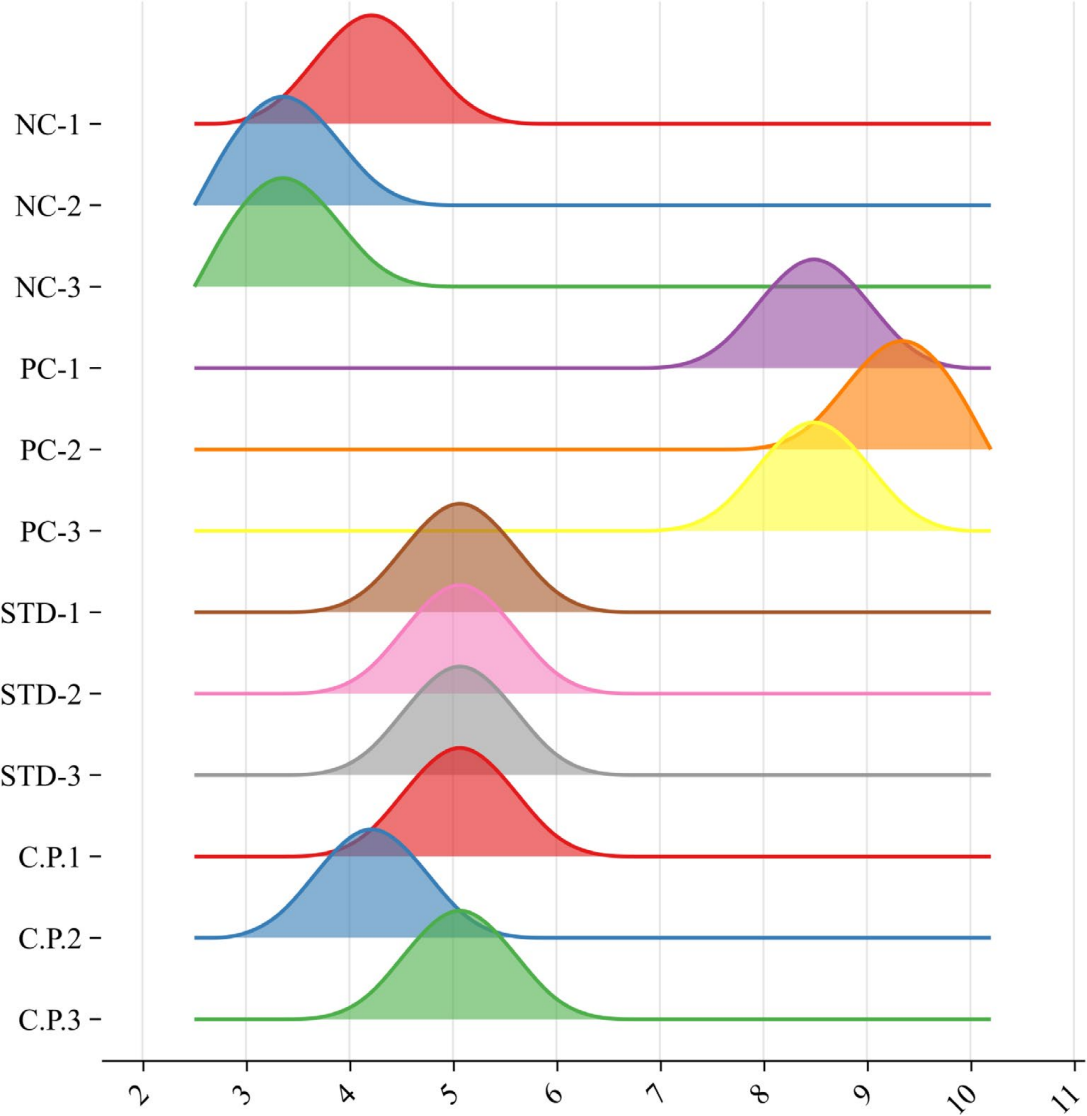
Scatter plot of Wet‐dry ratio with significant differences observed in the lungs across all groups.

To assess the incidence of pulmonary edema, the wet‐to‐dry weight ratio of the lungs was measured. This ratio was significantly higher in the PC group *(p ≤ 0.05)* compared to the NC group. Conversely, the wet‐to‐dry weight ratio was significantly lowered in the C.P and STD groups *(p ≤ 0.05)*, as illustrated in Figure 3.

### 3.5 Gene Expression Analysis (qRT‐PCR)

**FIGURE 4:**
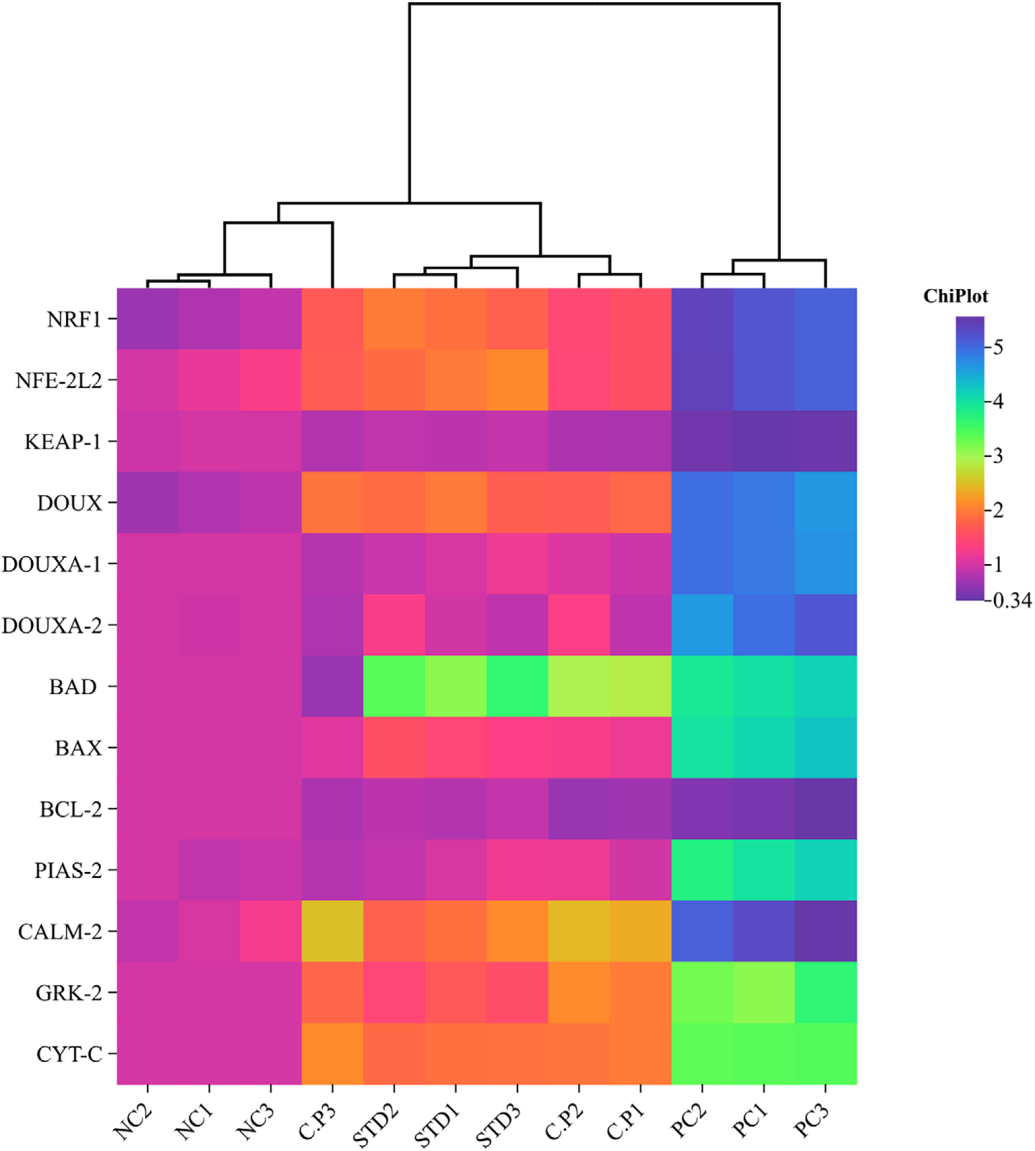
Heat map shows the up‐regulation and down‐regulation of DUOX, DUOXA‐1, DUOXA‐2, NRF1, KEAP‐1, NFE‐2 L2, BAD, BAX, BID, CASP‐9, BCL2, CYT‐C, PIAS‐2, CALM‐2, and GRK‐2 among different groups (created using online tool ChiPlot).

qRT‐PCR was performed to assess the regulation of gene expression (folds) in dual oxidases, calcium signaling, apoptotic, and NRF/KEAP‐1 pathways. The study investigated the molecular interaction of these genes in ALI and in maintaining the oxidative balance. Results showed that DUOX was significantly up‐regulated (three‐fold) in PC *(p ≤ 0.05)* as compared to the NC group. Whereas in C.P and STD groups, the expression level was downregulated significantly *(p ≤ 0.05)* in comparison to the PC, as shown in Figure 4. A similar trend in DUOXA1 was seen, with a significant difference with the NC group. On the other hand, the expression level in the treatment groups (STD and C.P) was downregulated significantly *(p ≤ 0.05)* as shown in Figure 4. Additionally, the activity of DUOXA2 was significantly upregulated in the PC group *(p ≤ 0.05)* compared to the NC group, and significantly down regulated in both treatment groups *(p ≤ 0.05)*, shown in Figure 4. KEAP‐1 and NRF1 reflect the redox homeostasis and antioxidant response; in this study, the genetic analysis showed that KEAP‐1 was significantly upregulated in the PC group *(p ≤ 0.05)* compared to the NC group. Whereas significant downregulated in the expression of KEAP‐1 was noticed in the C.P and STD groups *(p ≤ 0.05)*, shown in Figure 4.

Statistical analyses also revealed a significant up‐regulation (two fold) of NRF1 in the PC group *(p ≤ 0.05)* compared to the NC group. However, a significant increase in NRF1 expression was observed in both the C.P and STD groups *(p ≤ 0.05)*. The statistical analyses revealed a significant downregulation of NFE2L2 in the PC group compared to the NC group *(p ≤ 0.05)*. In contrast, NFE2L2 expression was significantly upregulated in both the STD and C.P groups *(p ≤ 0.05)*, suggesting activation of a compensatory antioxidant response (Figure 4). In the calcium signaling pathway, GRK‐2 expression in the PC group showed a significant upregulation as compared to the NC group *(p ≤ 0.05)*. However, GRK‐2 expression was significantly downregulated in STD and C.P groups *(p ≤ 0.05)*. Similar to GRK‐2, expression of CALM‐2 and PIAS‐2 was significantly upregulated in the PC group compared to the NC group *(p ≤ 0.05)*. However, in the treatment groups, CALM‐2 and PIAS‐2 expression was significantly downregulated *(p ≤ 0.05)*, shown in Figure 4.

This study investigated the expression of genes related to apoptosis and anti‐apoptosis, including BAD, BAX, BID, BCL2, CASP‐9, and CYT‐C, to elucidate the molecular mechanisms underlying experimental conditions. BAD expression was significantly upregulated in the PC group as compared to the NC group *(p ≤ 0.05)*. However, BAD was significantly downregulated in the STD and C.P groups *(p ≤ 0.05)*, potentially inhibiting the apoptotic pathway. BAX expression was significantly upregulated in the PC group compared to the NC group *(p ≤ 0.05)*; conversely, BAX was significantly downregulated in the STD and C.P groups *(p ≤ 0.05)*. BID, a pro‐apoptotic gene, was significantly upregulated *(p ≤ 0.05)* in the PC group compared to the NC group, while in the STD and C.P groups, BID expression was significantly downregulated *(p ≤ 0.05)*. BCL2 was significantly downregulated *(p ≤ 0.05)* in the PC group compared to the NC group; however, BCL2 expression was significantly upregulated in the STD and C.P groups *(p ≤ 0.05)*. CYT‐C expression was significantly upregulated in the PC group, indicating enhanced apoptotic signaling. In contrast, CYT‐C expression was significantly downregulated in the STD and C.P groups *(p ≤ 0.05)*, suggesting attenuation of apoptosis. CASP‐9 expression was significantly upregulated in the PC group compared to the NC group *(p ≤ 0.05)*; CASP‐9 expression was downregulated in the STD and C.P groups *(p ≤ 0.05)* (Figure 4). To summarize, these findings suggest that asbestos exposure led to an upregulation of pro‐apoptotic genes and a downregulation of anti‐apoptotic genes. The therapeutic potential of *C. papaya* may contribute to the restoration of oxidative balance and modulation of these apoptotic pathways.

### 3.6 Microscopic Insights

**FIGURE 5:**
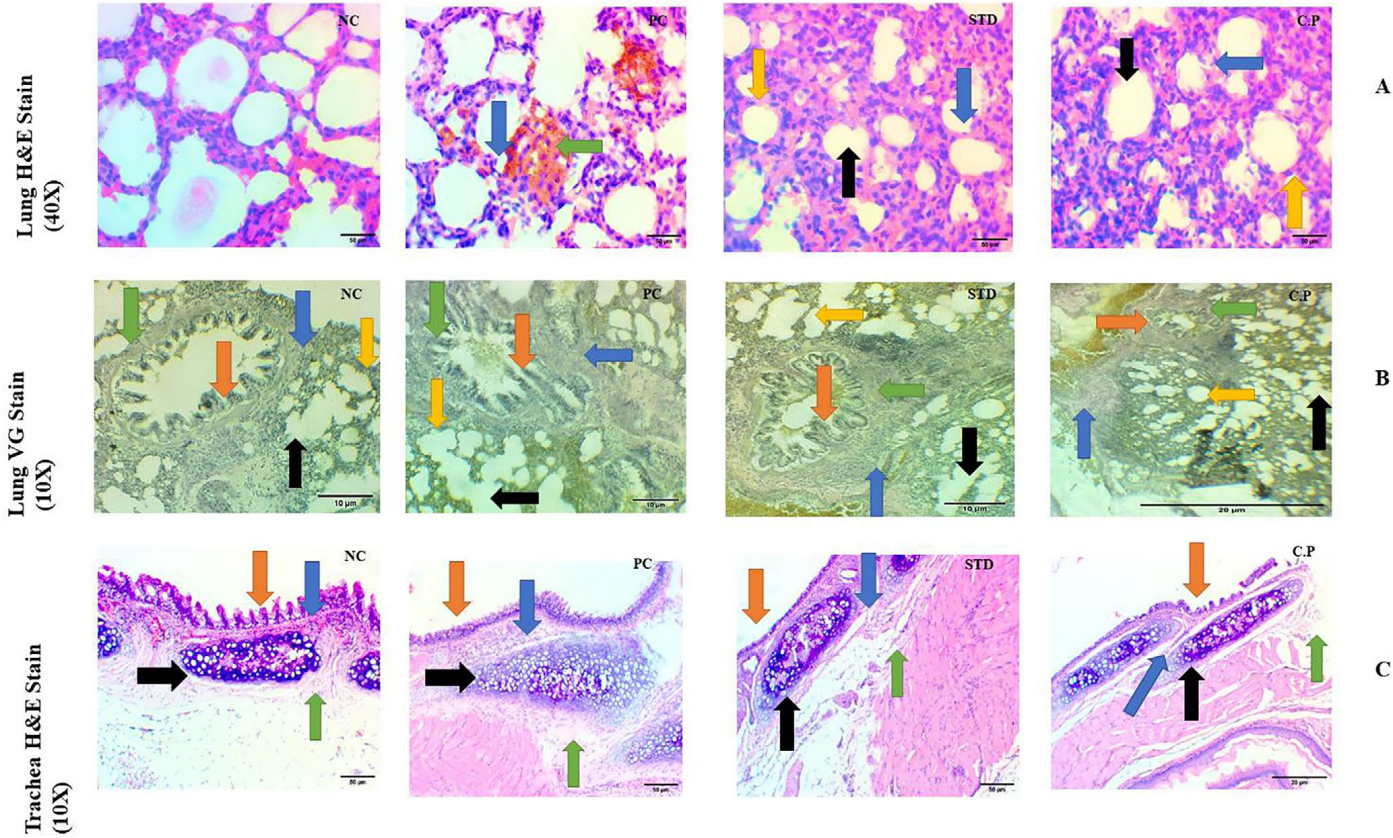
(A) Lung Hematoxylin & Eosin Stain (40X): Yellow arrows indicate the alveolar wall, black arrows point to alveoli, blue arrows mark the interalveolar septum, and green arrows show asbestos fiber accumulation in the NC, PC, STD, and C.P groups. (B) Lung Van Gieson Stain (10X): Yellow arrows depict alveoli, black arrows mark the alveolar sac, red arrows indicate the pseudostratified columnar epithelium, blue arrows highlight smooth muscles, and green arrows reveal the terminal bronchiole in the NC, PC, STD, and C.P groups. (C) Trachea Hematoxylin & Eosin Stain (10X): Orange arrows indicate ciliated epithelium, blue arrows mark sero‐mucous cells, black arrows point to hyaline cartilage, and green arrows display fibroblastic tissues in the NC, PC, STD, and C.P groups.

Lab‐grade Compound Light microscope (IRMECO GmbH & Co. Model IM‐910, Germany) with camera (TOUPCAM UCMOS14000KPA) attached with software (ToupviewX64) was used to record the photomicrographs. Histology of lung tissue (H&E stain 40X A) was recorded: The NC group showed normal parenchyma of the lung and structural organization, as illustrated in Figure 5A. In the PC group, the airways became narrower and thickened, with disruption of the alveolar wall, widening of the inter‐alveolar septum, and alveoli lodged with fibers of asbestos, as shown in Figure 5A. In contrast, the STD and C.P. groups exhibited parenchyma restoration, reduction in edema, repair of the alveolar wall, reduction in the thickened inter‐alveolar septum, and clearance of asbestos fibers, as depicted in Figure 5A. Lung tissue histology of the NC group (Van Giesons stain 10X B) presented the normal architecture of alveoli, alveolar sacs, pseudo‐stratified columnar epithelium, and maintained bronchiolar structure, as shown in Figure 5B. In the PC group, there was disruption of alveoli, ruptured alveolar sacs, sloughing of pseudo‐stratified columnar epithelium, distortion of terminal bronchioles, and hypertrophy of smooth muscle, as described in Figure 5B. In the STD and C.P. groups, there was reversion of bronchiolar structure and remodeling of alveoli and air sacs, as visible in Figure 5B. Histology of the trachea in the NC group (H&E stain 10X C) revealed intact ciliated epithelium, seromucous membrane, normal hyaline cartilage, and fibroblastic tissue, as manifested in Figure 5C. In contrast to the NC group, the PC group exhibited loss of cilia from the epithelium, damaged hyaline cartilage, and increased fibro‐elastic tissue, as shown in Figure 5C. The STD and C.P groups indicated a gain of ciliated structure, integrity of the membrane, and remodeling of hyaline cartilage and fibroelastic tissue.

## 4 Discussion

Asbestos is a major environmental carcinogen well known for triggering oxidative stress and cellular damage (Ali et al. 2025). *C. papaya* has gained attention due to its therapeutic potential in managing oxidative stress‐related conditions. This study aims to elucidate the molecular mechanism underlying its effects and to explore the role of *C. papaya* in reducing the asbestos‐induced acute lung injury. Oxidative stress is produced due imbalance in the ROS and the natural scavenging system of the body (Taha et al. 2025). Superoxide radicals are catalyzed into hydrogen peroxide and oxygen molecules by the superoxide dismutase's activity, whereas the catalase enzyme is responsible for dissociating hydrogen peroxide and mitigating oxidative damage and acute lung injury (Demirci‐Çekiç et al. 2022). These findings revealed an impaired antioxidant system in asbestos exposed group, as SOD and catalase were decreased in the PC group; conversely significant increase was noticed in the C.P group *(p ≤ 0.05)*. Results highlighted that the modulation of antioxidant enzymes was enhanced in the C.P group. GST enzymes detoxify various harmful substances produced during asbestos exposure, while the GPx enzyme has been involved in the reduction of hydrogen peroxide (Cai et al. 2024). In this study significant decrease in serum GST and GPX was observed in the PC group *(p ≤ 0.05)* as compared to the other groups due to the strong antioxidant potential of *C. papaya* . MDA is another indicator of oxidative stress, which causes damage to the lipid layer in the membranes and induces its peroxidation (Bonetta et al. 2024). In this study, due to oxidative damage and membrane disruption, MDA was significantly higher in the PC group than in the C.P group *(p ≤ 0.05)*. Due to the potential antioxidant activity of *C. papaya* , the oxidative damage and ROS were neutralized (Kim et al. 2014). This protective impact against ROS is attributed to its free radical scavenging system, and active phytochemicals, i.e., flavonoids, phenol, and alkaloids (Zinellu et al. 2021). Nitric oxide (NO) has a role in physiological and pathological processes, and it is a major contributor to inflammation and oxidative stress. Normally, it is essential for many signaling pathways, roles in blood vessels, and immunity (Janicka et al. 2024). Due to oxidative damage, NO level was elevated significantly in the PC group *(p ≤ 0.05)*; however, *C. papaya* leaves extract not only reduces the elevated level but also regulates the nitric oxide to carry on the physiological processes due to the presence of arginine and nutrient citrulline, which are the precursors for the production of the NO. LDH enzyme is present in muscle, lung, liver, and heart tissues. Current study showed *C. papaya* due to its antioxidants protection against stress helps tissues and cells to restore its damage as seen in C.P group. The exact pathway involved at the molecular level and antioxidant effect on lipids peroxidation needs to be understood yet. A critical parameter that is associated with the lung injury is wet‐to‐dry ratio an indicator of lung edema which is the hallmark of the lung injury (Bazzano et al. 2020). Damage due to asbestos exposure and as a result of inflammation, lung edema occurs, which enhances the permeability of the membranes and exudate accumulation in the interstitium of the lungs and space between the alveoli (Song et al. 2021). *C. papaya* has anti‐fibrotic activity that helps in reducing fluid leakage and accumulation, due to which it potentially mitigates the dry ratio of the lung tissue. Similarly, BALF analysis provided the number of macrophages and neutrophils, leakage of the protein from the disrupted epithelium, and an increase (Han et al. 2024). The barrier of the alveolar capillary is disrupted when protein from plasma pours into the space between the alveoli (Yue and Guidry 2019). Loss of homeostasis in the membrane permeability and oxidative stress‐dependent inflammation has increased the protein content (total protein and albumin protein) in the lungs (Van Hoecke et al. 2017). *C. papaya* leaf extract has attenuated the protein level and also maintained the endothelial and epithelial wall of alveoli integrity (Nguyen et al. 2013).

Permeability of epithelial membranes has been lost due to the inflammation and oxidative stress. Oxidative stress, a hallmark of inflammation that regulates multiple complex mechanisms, among them is KEAP‐1/NRF2 pathway that down‐regulates the oxidative damage (Schiavello et al. 2021). Under basal conditions, NRF2 is bound to KEAP‐1 in the cytoplasm; oxidative stress triggers dissociation of the NRF2‐KEAP‐1 complex (Vemula et al. 2024). When it translocates to the nucleus, it eventually activates some transcription factors and antioxidant response elements (ARE) (Biswas and Chan 2010). In the current study, the antioxidant effects of the leaf extract of *C. papaya* down‐regulated KEAP‐1 while up‐regulating the expression level of NRF2. In Pathological conditions, reactive oxygen species cause the modulation of the calcium channels. Increased calcium leakage in the cytosol can cause membrane disruption, production of the misfolded protein (stress in the endoplasmic reticulum), and alteration in the homeostasis of calcium due to dysfunction of the CALM‐2 (Sukumaran et al. 2021). The calcium signaling pathway and the NRF2 (a transcription factor) are regulated by these enzymes, which play a role in the generation of the oxidative material in the cell. This study indicated the activation of the calcium signaling pathway (like GRK, PIAS‐2, and CALM‐2), which lowered down the burden of oxidative stress (Palluzzi et al. 2017). The results of the current study show that *C. papaya* leaves extract causes the down‐regulation of the calcium signaling pathway. Oxidative damage causes the activation of the apoptotic proteins, and the release of CYT‐C leads to the death of the cell (Celep et al. 2024). BAX and BAD are responsible for apoptosis; they trigger the breakage of the membrane of the mitochondria and the reflux of factors that promote apoptosis CYT‐C (Qian et al. 2022). BCL2 is known for the anti‐apoptotic properties that reside in the membrane of the mitochondria. When BCL2 is activated this stops the Cytochrome C release and inhibits apoptosis and cell death (Tutuncu and Ozdemir 2023). During oxidative stress, BCL2 activity is restricted due to its binding with the BAD protein. In this study, *C. papaya* leaves extract increased permeability of the membranes, down‐regulation of apoptotic genes, i.e., BAD, BAX, and BID (Angwa et al. 2022), and stimulation of BCL2 and CYT‐C (Alam et al. 2022). Impaired and deregulated dual oxidases have been linked to many disorders, such as thyroid, immune, and inflammatory disorders (Damiano et al. 2012). DUOX, DUOXA1, and DUOXA2 belong to the members of the NADPH oxidase (Luxen et al. 2009). Dual oxidases have a crucial role in hydrogen peroxide production, maintain epithelial permeability, and prevent pathogen colonization. It helps in redox signaling, provides defense on the mucosal surface of the GIT and respiratory tract, and synthesizes tri‐iodothyronine and tetra‐iodothyronine (Luxen et al. 2008). The upregulation of DUOX during pathological conditions causes oxidative stress (Khan et al. 2025). This research highlighted the significance of *C. papaya* as a potent inhibitor of dual oxidase enzymes due to its antioxidant activity, as significantly seen in the C.P group. The significant decline in the dual oxidase enzymes strengthens previous study on oxidative stress (Shakak et al. 2025). Histology of the lung NC Group showed normal parenchyma of the lung and structural organization. In the PC group, airways become narrower and thickened with destruction of the alveolar wall, and dense with asbestos fibers (Punsmann et al. 2021). Lung Van Gieson Stain tissue histology of the NC group showed normal architecture of alveoli and maintained bronchiolar structure. In the PC group, there is destruction of alveoli, sloughing of pseudo‐stratified columnar epithelium, and distorted terminal bronchiolesalong with reversion of bronchiolar structure and remodeling of alveoli and air sacs (Zakaria et al. 2021; Abdelhiee et al. 2025).

## 5 Conclusion

This study demonstrates the potential of the *C. papaya* as a natural therapeutic agent that enhances the activity of the antioxidant defence system by restoring the antioxidant enzymes and lipid peroxidation. *C. papaya* reduces oxidative stress, stimulates the anti‐oxidant response of the cell, and lowers the edema and total protein. Molecular analysis revealed that *C. papaya* may be involved in the modulation of calcium signaling, NRF2‐mediated antioxidant pathways, and apoptosis‐related signaling cascade in lung damage. *C. papaya*, due to its potent scavenging potential, down‐regulates gene expression, improves the parenchyma of the lungs, and facilitates removal of asbestos fibers from the alveoli. The findings of current study provide a foundation for further investigations to mitigate oxidative stress and attenuating lung injury.

## 6 Limitations & Future Recommendations

Several limitations of the present study warrant consideration. First the exposure regimen was designed to induce sub‐chronic inflammation and oxidative injury without causing motality. While appropriate for therapeutic evaluation, it may not fully replicate the chronic, progressive nature of human asbestos related pulmonary disease. Second, although the rat model provides well established preclincal evidence, interspecies differences in drug metabolism, immune response, and tissue repair mechnism necessitate caution when extrapolating these findings to humans. Future investigations should focus on dose optimization and pharmacokinetic profiling to guide translational development. Additionally long‐term exposure models and well controlled trials are required to assess the efficacy, safety, and the therapeutic relevance of *C. papaya* extract in human population. The transcriptional evidence presented herein provides a robust molecular foundation for future research.

## Author Contributions

**Usman Haider:** writing – original draft, conceptualization, methodology, software, investigation. **Muhammad Ahmad:** writing – review and editing, investigation, validation, software, methodology. **Wania Nasir:** writing – review and editing, formal analysis, investigation. **Najeeb Ullah Khan:** software, formal analysis, methodology. **Muhammad Saad Tariq:** investigation, visualization. **Bilal Aslam:** supervision, resources. **Saif Ur Rehman:** supervision, writing – review and editing. **Junming Sun:** supervision, resources.

## Funding

This work was supported by Key Research and Development Plan of Qingxiu District Science and Technology Bureau, Nanning City (2021011); The Funding Science Foundation of China‐ASEAN Laboratory Animal Science and Technology Innovation Center, Guangxi Medical University (KCZX2024003).

## Conflicts of Interest

The authors declare no conflicts of interest.

## Data Availability

The data that support the findings of this study are available from the corresponding author upon reasonable request.
